# Mindfulness for children with ADHD and Mindful Parenting (MindChamp): Protocol of a randomised controlled trial comparing a family Mindfulness-Based Intervention as an add-on to care-as-usual with care-as-usual only

**DOI:** 10.1186/s12888-018-1811-y

**Published:** 2018-07-25

**Authors:** Nienke M. Siebelink, Susan M. Bögels, Lisanne M. Boerboom, Noor de Waal, Jan K. Buitelaar, Anne E. Speckens, Corina U. Greven

**Affiliations:** 1 Department of Cognitive Neuroscience, Donders Institute for Brain, Cognition and Behaviour, Radboud University Medical Centre, Nijmegen, The Netherlands; 2 Karakter Child and Adolescent Psychiatry, University Center, Reinier Postlaan 12, Nijmegen, 6525 GC The Netherlands; 3 Research Institute of Child Development and Education, University of Amsterdam, Amsterdam, the Netherlands; 4 Department of Psychiatry, Radboudumc Centre for Mindfulness, Radboud University Medical Centre, Nijmegen, The Netherlands; 5 King’s College London, Social, Genetic and Developmental Psychiatry, Institute of Psychiatry, Psychology and Neuroscience, London, UK

**Keywords:** Self-control, Executive function, Attention, Adolescent, Parents, Mindfulness, ADHD

## Abstract

**Background:**

Self-control in childhood has been linked to long-term and cascading effects on health, academic, criminality, wealth and parenting outcomes. Hence it is important to target self-control deficits early in life. Self-control deficits are a hallmark of Attention Deficit/Hyperactivity Disorder (ADHD). Even after receiving care-as-usual (CAU) for ADHD, impaired self-control often remains. Pharmacotherapy can be hampered by side-effects, low adherence and short-term effectiveness. Other limitations of CAU are decreased effectiveness when parents have ADHD and little effect on parental well-being. Mindfulness-Based Interventions (MBIs) are an emerging non-pharmacological approach with potential to improve self-control and well-being in both children and parents. However, there is a lack of sufficiently powered randomised controlled trials (RCTs) to establish their effects in families with ADHD. This study protocol describes an RCT to investigate the effectiveness of a family MBI as an add-on to CAU in treatment of youth with ADHD, and is described in accordance with Standard Protocol Items: Recommendations for Interventional Trials (SPIRIT).

**Methods/design:**

An RCT will be conducted in *N* = 100 children (aged 8–16 years) with ADHD and their parents. The experimental condition will consist of a family MBI (MYmind): 8-week group-based MBI for youth combined with parallel group-based Mindful Parenting for their parents, as an add-on to CAU. The control condition will consist of CAU-only. Assessments will take place at baseline, end of treatment (3 months later), 2 and 6 months’ follow-up. Primary outcome measure will be an ecologically valid assessment of child self-control with the parent-rated Behaviour Rating Inventory of Executive Function (BRIEF). Secondary child outcome measures will be teacher-rated BRIEF, computerised self-control tasks and questionnaires on psychological symptoms (e.g. ADHD, symptoms of autism), well-being and mindfulness. For parental outcomes, secondary measures will be self-rated BRIEF, computerised self-control tasks and questionnaires on psychological symptoms, well-being and mindful parenting.

**Discussion:**

The proposed RCT will take account of methodological limitations of previous studies on MBIs in child ADHD populations. The current study will provide valuable information on family MBI as a potential effective intervention in targeting self-control deficits for youth with ADHD and their parents.

**Trial registration:**

ClinicalTrials.gov NCT03220308. Retrospectively registered 18 July 2017.

**Electronic supplementary material:**

The online version of this article (10.1186/s12888-018-1811-y) contains supplementary material, which is available to authorized users.

## Background

Self-control can be seen as an umbrella construct that bridges concepts from different disciplines (e.g., executive function, impulse control, attention-regulation, emotion-regulation, planning, delay of gratification, and cognitive flexibility) [1, 2]. Poor self-control is an important feature of psychiatric disorders arising in childhood and adolescence, including Attention Deficit/Hyperactivity Disorder (ADHD), oppositional defiant disorder (ODD), conduct disorder (CD), mood and anxiety disorders, substance use disorder, and gambling [3–5]. Early self-control predicts not only mental and physical health, but also current and later academic performance, wealth, criminality and parenting [1, 6–8]. For example, children with poor self-control at preschool age have been shown to be more likely to have substance dependence, financial troubles and a criminal record at age 32 [1]. Therefore, targeting self-control in interventions for youth with deficits in self-control appears of utmost importance.

Self-control can be trained, although it is unclear which interventions are most effective in improving self-control in youth [9]. Mindfulness-Based Interventions (MBIs) use exercises that train self-control capacities [10] and are increasingly gaining ground as an approach to increase self-control in youth [11, 12]. Mindfulness is often defined as the trainable capacity to pay attention to experiences in the present moment, on purpose and without judgement [13, 14]. It has been suggested that MBIs enhance self-regulation through three interacting processes: enhanced attention control, improved emotional regulation, and altered self-awareness (diminished self-referential processing and enhanced body awareness) [15]. This is supported by an activation likelihood estimation meta-analysis of 21 structural neuroimaging studies in ~ 300 adult (mindfulness) meditation practitioners, showing that neural correlates of these cognitive processes are altered in meditators compared to non-meditators with a global medium effect size [16]. In addition, 78 functional neuroimaging studies were reviewed in an activation likelihood estimation meta-analysis with 527 healthy non-clinical adult meditation practitioners who actually meditated during scanning. The results indicate dissociable brain (de)activation patterns during different styles of meditation, which are congruent with the psychological and behavioural aims of each practice [17]. If neural mechanisms underlying self-control can be altered structurally and functionally by meditation practices, this likely results in effects on behavioural and cognitive measures of self-control as well.

Previous clinical trials show that self-control can be improved following MBIs in diverse populations. Randomised Controlled Trials (RCTs) show greater improvement after MBIs than active control conditions in executive functions of adults (undergraduates, *N* = 80), adolescents (middle and high school students, *N* = 489) and children (4–5 years, *N* = 60) [18]. A meta-analysis on neuropsychological outcomes of MBIs in adult populations (clinical and non-clinical) found preliminary evidence for positive effects on general awareness and meta-awareness, cognitive flexibility and working memory [19]. However, evidence was weak for improvement of attentional control (alerting/sustained attention, orienting/selective attention and executive attention), executive function inhibition and mental set shifting. For youth (5–18 years old), a systematic review of interventions with a focus on yoga-, meditation-, and/or mindfulness-based techniques found significant effects of the interventions on attention and executive functioning with medium to large effect sizes in five of the thirteen included studies. However, study populations consisted of both non-clinical and clinical populations and methodological quality of the studies varied [20]. A meta-analysis of MBIs with youth found significantly higher effect sizes for clinical samples than for non-clinical samples [21]. In addition, an RCT found that specifically children with poor executive functioning show improvement in this area based on teacher and parent reports [22]. Hence, effectiveness of MBIs in increasing self-control may be greater in a more homogeneous clinical population with self-control difficulties.

As a consequence, MBIs are increasingly applied as an approach in the treatment of ADHD in which deficits in self-control are a central component [23]. ADHD is a common neurodevelopmental disorder characterised by impairing symptoms of inattention and hyperactivity-impulsivity, affecting 5–7.5% of all children worldwide [24]. The annual health care costs and societal ‘costs of illness’ of ADHD in youth are high and comparable in magnitude to other serious medical problems (e.g. stroke, asthma in children) and mental health problems (e.G. *major* depressive disorder) [25]. Furthermore, ADHD has a significant impact on the quality of life of the affected children comparable to other mental health conditions (e.g. anxiety disorders, autism spectrum disorders (ASD), mood disorders) and severe physical disorders (e.g. cerebral palsy, cancer) [26, 27]. Moreover, quality of life of parents is also negatively affected by ADHD of their child, for example in terms of their psychological well-being, personal fulfilment, family and couple relationships and daily life activities [28].

Current care-as-usual (CAU) for children with ADHD consists of psychoeducation, pharmacotherapy and/or (cognitive-) behavioural treatments [29, 30]. Psychoeducation enhances parents’ knowledge about ADHD and may enhance engagement in psychopharmacological treatment [31]. Pharmacotherapy can be effective in reducing core symptoms of ADHD and to a lesser extent improving other outcomes like quality of life, functioning [32], response inhibition, sustained attention and working memory [33]. However, pharmacotherapy can be hampered by side effects and low adherence [34–38]. Meta-analyses of (cognitive) behavioural interventions in the treatment of youth with ADHD show that these can improve parenting behaviour, increase parents’ sense of self-worth and reduce child conduct problems. Specific modules may improve child social skills and academic functioning [31]. Nevertheless, this review also shows that behavioural treatment does not reduce observer rated ADHD symptoms of the child and no support was found for its effects on parental mental well-being [31, 39]. Moreover, cognitive training of attention and executive functioning in children with ADHD does not yield significant improvements in these areas [40]. Another limitation of both pharmacotherapy and behavioural interventions for ADHD is that treatment is less effective when parents have ADHD themselves [31, 41]. As ADHD runs in families and is highly heritable, this is often the case [42, 43]. In conclusion, CAU for ADHD is not sufficient for many families and a substantial subgroup of children with ADHD has remaining symptoms and impairment [44, 45]. MBIs for children and their parents are promising in targeting self-control, behavioural symptoms and parental outcomes [46–49].

Previous studies on MBIs as a treatment approach for youth with ADHD have been reviewed in several publications. A meta-analysis of studies on pre-post effects of MBIs on ADHD core symptoms in children and adults diagnosed with ADHD [50] yields an overall effect size of d = −.66 for reduction of inattention symptoms and d = −.53 for reduction of hyperactivity/impulsivity symptoms. In a subgroup analysis of the six included trials in children and adolescents only, medium effect sizes were calculated for both inattention (d = −.66) and hyperactivity/impulsivity (d = −.47). Next to effects on ADHD core symptoms, other clinical effects in children with an ADHD diagnosis (≤ 18 years) and their parents were studied in a systematic review which included a broader range of interventions, i.e. studies with an intervention focusing on mindfulness and/or yoga techniques with either parents, children and/or parents plus children [51]. Eight of the sixteen studies targeted children only and another eight studies investigated a family-based intervention. Positive results were found for improvements after meditation-based interventions in parent-reports of child functioning, parent and/or teacher-reports of child internalising/externalising behaviour, parental ADHD symptoms, parental satisfaction, parent–child relationships and parental happiness, but not in child self-reported happiness. Mixed or limited evidence was found for improvements in child ADHD symptoms, child self-esteem, child social functioning, child academic performance, child self-report of functioning, child-report of internalising/externalising symptoms and parental stress and over reactivity of the parent. Finally, larger effect sizes in child outcomes, lower incidence of poorer outcomes, and more favourable outcomes for parents resulted from trials with family-based interventions compared to child-only interventions. In conclusion, previous research on the effectiveness of MBIs in the treatment of youth with ADHD shows these are feasible interventions for ADHD with potential benefits across a broad range of outcomes including ADHD symptoms, well-being and outcomes for parents. However, the results above should be interpreted with caution due to limited methodological quality of the reviewed studies. It is of note that none of the systematic reviews on MBIs for youth with ADHD focused on self-control in particular.

A few clinical trials looked at effects of MBIs on self-control as assessed with neurocognitive tests and/or with questionnaires in youth with ADHD. In a first RCT, comparing a family MBI with a waitlist control group in children with ADHD aged five to seven years (*N* = 100) and their parents, significantly greater improvement in the family MBI group compared to control group was found for child executive attention (conflict monitoring)(d = .41) [52]. This result is in line with a quasi-experimental trial on neurocognitive task performance following an MBI in adolescents (*N* = 8) and adults (*N* = 24) with ADHD, which also found significant improvements for measures of executive attention. In addition, significant effects were found for set-shifting [53]. A pilot pre-post-intervention study with a family MBI in children aged eight to twelve years (*n* = 11) found significant improvement with large effect sizes on objective attention tests, but not on parent ratings of self-control on the Behaviour Rating Inventory of Executive Function (BRIEF) [54]. In contrast, in a quasi-experimental trial with ten adolescents (aged 11–15 years) following a family MBI, self-control assessed with the BRIEF improved significantly at 8-weeks follow-up, but only according to father reports and not mother reports [55]. For youth with ADHD, no RCT has been published on effects of MBIs on ecologically valid questionnaire ratings of self-control (e.g. BRIEF). In adults with ADHD, a randomised waitlist controlled MBI trial (*N* = 20) resulted in significant group differences at the end of treatment favouring the MBI group on self-reported self-control (assessed in the laboratory and with ecological momentary assessment) and clinician rated self-control with large effect sizes. In contrast, no significant improvement was observed with the objective self-control tasks [56]. In another waitlist RCT on an MBI in adults with ADHD (*N* = 103), self-control as assessed with the BRIEF improved with a large effect size (d = .93) as well [57]. Furthermore, in a randomised CAU controlled MBI trial in 120 adults with ADHD, improvement on the BRIEF over time after MBI + CAU compared with CAU only was found, resulting in an effect size of d = .49 at 6-month follow-up [58]. In summary, implementing MBIs in the treatment of youth with ADHD to improve self-control is promising. Although there are preliminary positive results, the existing evidence in youth is insufficient due to a lack of studies with good methodological quality. RCTs with sufficient power are needed.

The current protocol describes an RCT comparing an 8-week family MBI for youth with ADHD and their parents (MYmind) in addition to CAU with continuation of CAU only. Participating children will have an ADHD diagnosis and comorbidities will be allowed (including ASD). The primary, secondary and tertiary aims correspond to the primary, secondary and tertiary measures that will be used in this study. The primary aim is to investigate the effectiveness of a family MBI in improving self-control of youth with ADHD, as assessed with ecologically valid parent-ratings (BRIEF-P, primary outcome). Different definitions are used in literature on self-control, some take a broad approach, other narrow, and opinions vary on what self-control comprises. We take a broad view on self-control in that it entails self-regulation of behaviour, emotion, cognition and attention. Given the moderate convergent validity of neurocognitive tests of self-control, we prefer ecologically-valid questionnaire ratings of self-control over neurocognitive tests [2]. The predictive validity of behavioural questionnaire ratings of self-control on clinically and societally relevant objective outcomes even decades later (e.g. months unemployed, single parenthood, criminal conviction) has been documented [1, 59]. The secondary aim is to examine the effects of family MBI on child self-control as assessed with teacher-ratings (BRIEF-T) and objective computerised tasks and on psychological symptoms (e.g. ADHD symptoms, symptoms of ASD, brooding), well-being and mindfulness of the children (secondary outcome measures for children). In addition, we aim to examine effects of family MBI on parental self-control as assessed with ecologically valid self-ratings (BRIEF-A) and objective computerised tasks and on psychological symptoms, well-being and mindful parenting of the parents (secondary outcome measures for parents). Our tertiary aim is to look at the effects of family MBI on some exploratory measures such as mind-wandering. Finally, child saliva samples will be collected for (epi)genetic research and qualitative data will be collected to explore effects that are not captured with quantitative assessments and to explore facilitators and barriers of family MBI for youth with ADHD and their parents.

## Methods/design

### Study design

This is a parallel group RCT in which all participants with ADHD receive CAU. Randomisation will assign children with ADHD and their parents to either a family MBI (plus continuation of CAU) or to the control condition (continuation of CAU) with a 1:1 ratio.

### Participants

The study population will consist of children aged 8–16 years with an ADHD diagnosis and at least one of their parents. The families will be recruited through the outpatient clinic of Karakter, an institute for child and adolescent psychiatry. Recruitment will take place at three different Karakter locations in the central eastern part of the Netherlands, namely Nijmegen, Arnhem and Ede (see also the clinical trial registration). In addition, families will be recruited by media advertisements.

#### Eligibility criteria

We will include families who meet the following criteria: a) child is 8–16 years old and in the third grade of primary school or higher; b) child has a primary diagnosis of ADHD according to the Diagnostic and Statistical Manual of Mental Disorders (DSM-IV or DSM-5) system [60, 61] and confirmed by a structured interview (see Assessments section); c) child receives CAU for ADHD and has remaining ADHD symptoms (average score > 1.0 on the investigator-rated DSM-5 items from the Conners’ ADHD rating scale [62]); d) ADHD medication dose of child is stable, at least two weeks prior to baseline, or an informed decision is made on not taking ADHD medication; e) at least one parent is willing to participate. Exclusion criteria are a) psychosis, bipolar illness, active suicidality, untreated posttraumatic stress disorder or substance use disorder of the child that impedes functioning; b) psychosis, bipolar disorder, active suicidality, untreated posttraumatic stress disorder or substance use disorder of the parent that impedes functioning; c) child and/or participating parents have an estimated Intelligence Quotient (IQ) < 80; d) child and/or participating parents do not have adequate mastery of Dutch language; e) child and/or participating parents have participated in an > = 8-week mindfulness programme in the past year or ever in a Mindful Parenting training; f) child or parents participate in another intervention study.

### Intervention

#### Family MBI (MYmind)

We will use the MYmind course [63], a standardised, highly-structured protocol developed to target the specific difficulties that children with ADHD and their parents may confront when meditating. As self-control problems in regulating attention, impulses and motor activity are core issues of children with ADHD, the course is designed around these difficulties. The aim of MYmind is to teach children to meditate and to practice non-reactivity, that is, awareness of their impulses but not following them automatically. The course for the parents focuses on their role as a parent, through teaching them mindful parenting (e.g., to help guide their child with the meditations, practice non-reactive parenting and mindful communication with the child), but also directly addresses parents’ own self-control and behaviour.

MYmind consists of eight weekly 90-min group sessions for children (groups of approximately 5–8), and parallel group sessions for their parents of equal length and duration. The child and parent sessions take place in separate rooms, with the exception of sessions 1, 5 and 8, where part of the session is together in the same room. In the sessions (playful) mindfulness exercises are done and education is given around the following themes, one per session: 1) Beginners’ mind, 2) Home in our body, 3) The breath, 4) Distractors!, 5) Stress, 6) High way, walking way, 7) Acceptance & autonomy, and 8) The future. Both children and parents receive daily homework during the eight weeks of training (15 min for child and 30–45 min for parent, 6 days/week). Theory and exercises are described in a workbook that both children and parents receive together with audio-files to guide the mindfulness exercises. As children with ADHD may have motivational problems, a reward system is incorporated in the course for the children to enhance motivation to practice in the sessions and at home. The 8-week course is followed by eight weeks of home self-practice, and a single joint child-parent 90-min booster session. In the booster session, participants share experiences of the mindfulness practice in the last eight weeks where they did not have the support of the group training, rehearse certain practices in order to remind the families of the possible joys and benefits of mindfulness practice, and are encouraged to think about how to implement mindfulness in their lives and to renew their intentions. Participants who attend four or more sessions will be considered as ‘completers’.

The first trial on an MBI for children combined with Mindful Parenting was conducted by Bögels et al. (2008) [64], which resulted in the MYmind training. Since then, several non-randomised pilot studies have been conducted with the MYmind training in child and adolescent samples with ADHD [54, 55, 65–67] and with ASD [68, 69]. Findings are promising and show that the MYmind training is feasible and acceptable [70].

#### Mindfulness teachers

Mindfulness teachers will have to be experienced and meet quality criteria for category 1 from the VMBN (Association of mindfulness-based teachers in the Netherlands and Flanders), which are in accordance with the ‘UK Mindfulness-Based Teacher Trainer Network’ [71]. The criteria for category 1 of the VMBN include a college or university degree; > = three years of regular meditation experience; > = one 10-day retreat or two 5-day retreats in the past four years; having participated in a Mindfulness-Based Stress Reduction (MBSR) [72] or Mindfulness-Based Cognitive Therapy (MBCT) [73] course; > = 150 h of education in MBSR/MBCT (skills training in giving formal and informal practices, theory and practice of psycho-education and inquiry, theoretical foundation, supervision, reflection report, adequate use of course materials and experience in giving training); > = seven days in-service training per two years; giving > = two MBSR/MBCT courses per two years. In addition, for the current study, mindfulness teachers will be trained thoroughly in the administration of the MYmind protocol by following a 5½-day advanced teacher training in MYmind given by its developer (SB). Following an advanced teacher training in MYmind also gives teachers access to the course materials (i.e. workbooks and audio files) for the participants. During the trial, the mindfulness teachers will be supervised by SB who has extensive expertise and experience working with the intervention delivered in this proposal. In the child-group there will be a mindfulness co-teacher, who will have experience with children with ADHD, in addition to the mindfulness teacher. All sessions during the 8-week training will be videotaped for treatment integrity purposes. Mindfulness teacher competency will be assessed on the Teaching Assessment Criteria (MBI:TAC; [74, 75]) by experienced mindfulness teachers using a random selection of the videotapes on the Teaching Assessment Criteria (MBI:TAC [74, 75]).

#### CAU

According to the Dutch Multidisciplinary guidelines for the diagnosis and treatment of ADHD [76], CAU for children aged 8–16 years consists of psycho-education and the prescription of medication approved for ADHD and/or evidence-based parent and/or teacher-administered behaviour therapy, preferably both medication and behaviour therapy. First-line option for medication is a psychostimulant, second-line options are atomoxetine or alpha-2 presynaptic agonists. Participants in the CAU-group will be informed that it is not allowed to participate in a mindfulness programme until the 2-month follow-up assessments of the study.

#### Expectancy, satisfaction, compliance and healthcare consumption

For family MBI and CAU condition, credibility and expectancy of clinical outcome will be evaluated with the parent-rated 6-item Credibility/Expectancy Questionnaire (CEQ, translation to Dutch by the authors for this study) [77]. The CEQ shows a good internal consistency and test-retest reliability [77]. Satisfaction with family MBI/CAU will be assessed in parents and children at end of treatment, 2- and 6-month follow-up. Parental and child compliance with the mindfulness practices, and child adherence with any ADHD medication, will be assessed in the parents at the same three time points. Type and dose of possible ADHD-medication and other medication will be assessed at all time points for parent and child. At baseline parents will report which treatments for ADHD the child received prior to study participation. In addition, child healthcare consumption between assessments will be assessed with a 30-item adapted version of the parent-rated Trimbos and iMTA questionnaire on Costs associated with Psychiatric illness (TiC-P) [78] at end of treatment and both follow-up time points. Parental healthcare consumption during the whole study period will be assessed with the 46-item self-rated TiC-P at 6-month follow-up. The TiC-P is a valid questionnaire with a sufficient to high test-retest validity (ICC > .6) [79].

### Assessments

#### Descriptives

Clinical ADHD diagnosis will be confirmed using a structured psychiatric interview (Schedule for Affective Disorders and Schizophrenia for School-Age Children-Present and Lifetime Version; K-SADS-PL) [80], adapted to DSM-5 and administered to a parent by a trained researcher. The focus of the interview will be on the behaviour of the child off medication in the past half year. Further, parents will be screened for the presence of adult psychiatric disorders, using the 10-item Kessler Psychological Distress Scale (K10) [81]. For those with elevated scores (25 or higher) a diagnostic interview (Mini-International Neuropsychiatric Interview; MINI) [82] will be administered to establish axis-I disorders by a trained researcher. In case no valid IQ test results of participants are available, full-scale IQ will be estimated by two subtests of the Wechsler Intelligence Scale for Children-Third Edition (WISC-III) [83] or Wechsler Adult Intelligence Scale-Third Edition (WAIS-III) [84]: Vocabulary and Block Design. These subtests together are known to correlate between .88–.91 with the Full-scale IQ [85, 86]. Standardised questionnaires completed by parents as part of routine intake procedures at Karakter will give us a wide range of additional background information on variables such as age, gender, medical/medication history, ethnicity, family-structure and socioeconomic status. Some additional participant characteristics will be collected at baseline as there might be changes in family-structure or socioeconomic status between intake at Karakter and study participation. The Pubertal/Physical Development Scale (PDS) [87] will be used to assess the pubertal developmental stage of the child at baseline and 6-month follow-up.

#### Primary outcome measure

##### Parent-rated child self-control

To measure self-control of the child, we will use the parent-rated version of the BRIEF [88], which assesses real-life self-control skills across situations and is used in clinical and research settings for ADHD. The questionnaire consists of 75 items with a 3-point rating scale ranging from 1 (*never*) to 3 (*often*) with higher scores indicating more problems in executive functioning. The BRIEF gives rise to a global executive composite score as well as two broad indexes (Behavioural Regulation and Metacognition) and eight subscales (Inhibit, Shift, Emotional Control, Initiate, Working Memory, Plan/Organise, Organisation of Materials, and Monitor). The internal consistency of the Dutch version is sufficient when administered to adolescents with ADHD (Cronbach’s α coefficients ranging from .69 to .95) [55]. Test-retest reliability is high with intra-class correlations of ≥.73. The BRIEF demonstrates good convergent validity [88].

#### Secondary outcome measures for children

##### Teacher-rated child self-control

Child self-control will also be assessed using teacher-ratings on the BRIEF-T [88]. Teachers have the advantage to be able to evaluate the child’s behaviour in the context of their peers’ behaviour and of the normative range, and are blinded to exactly which intervention is provided to the child at home and/or in the clinic [89]. The BRIEF-T consists of 75 items with the same 3-point rating scale and structure as the parent form. The Dutch version shows a good internal consistency, with Cronbach’s α coefficients ranging from .88 to .98, and a high test-retest reliability (ICC = .77 for the global executive composite score) [88].

##### Computerised tasks of child self-control

Logan’s Stop Signal task will be used to measure motor inhibition [90, 91]. In this task, participants are presented with go-trials which consist of the presentation of a stimulus (i.e. either the letter O or the letter X) and are instructed to press the corresponding response button as quickly and accurately as possible. In 25% of the trials, at random, the go-trial is followed by an auditory stop-signal (i.e. a tone). In those trials, participants are required to withhold their response. The delay between the go and the stop signal is varied using a dynamic tracking algorithm, such that an average person has a 50% chance of correctly withholding their response at a stop-trial. The two main dependent behavioural measures will be the percentage of errors and the Stop Signal Reaction Time (SSRT), which is calculated by subtracting the Mean Delay from the Mean Correct Reaction Time [90]. Secondary behavioural outcome measures will be reaction time variability and speed accuracy trade off [92]. The Stop Signal task shows an adequate test-retest reliability when administered to children with ADHD (ICC = .72 for the SSRT) [91].

Temporal discounting refers to the decrease of subjective reward value as a function of increasing delay [93]. In the Temporal Discounting task that will be used in this study [94, 95] participants are asked to choose between a smaller immediate reward (i.e. one, two, three of four eurocents) and a larger delayed reward (i.e. five eurocents) during forty trials. Choices are visually represented by two airplanes on a computer screen. Delays are represented by the “height” at which the planes are flying, and vary between five and sixty seconds. Participants choose the preferred plane which results in earning the chosen amount, immediately or after the appropriate delay. A maximum of two euros can be earned. The computer task will be followed by a debriefing on the child’s choices and emotions regarding every delay interval using the Self-Assessment Manikin. This measure is shown to be a reliable, non-verbal method for assessing a person’s emotional reaction in response to a certain event or stimulus [96]. Outcome measure will be the subjective reward value, defined as the magnitude of the smaller immediate reward that leads to indifference of the participant towards the larger delayed reward [93]. At this point, the participant shows no clear preference for one of the rewards, either the delayed or the immediate one. The real Temporal Discounting task (with actual money and delays) has a clear advantage compared to hypothetical tasks in terms of ecological validity [97] and is proven useful in assessing individual differences in children versus adolescents [94] and individuals with less and more hyperactivity/impulsivity symptoms [98].

The Probabilistic Reversal Learning task will be used to determine the cognitive ability to adapt behaviour according to changes in stimulus-reward contingencies, also known as cognitive flexibility [99]. An experimental study has shown impaired reversal learning to reflect reduced inhibitory control of affective responses, and therefore might be related to deficits in self-control [100]. In the Probabilistic Reversal Learning task participants repeatedly choose between two visual stimuli during eighty trials, followed by rewarding or punishing auditory feedback. In 80% of the trials, the choice is followed by actual feedback (i.e. rewarding tone with correct response and punishing tone with incorrect response) and in 20% by misleading feedback. The stimulus-outcome contingencies reverse unannounced after forty trials, resulting in the previous mostly rewarded stimulus now being mostly punished and vice versa. Subjects are instructed that the contingencies may change, but do not know when or how often. Thus, the difficulty in performing the task comes from the need to integrate feedback over a number of trials because negative feedback may either be a probabilistic punishment or signal a true reversal. Outcome measures will be Ratio Win-Stay (the number of times the participant repeats their choice following a rewarded trial, divided by the total times the participant repeats their choice), Ratio Lose-Shift (the number of times the participant shifts response following a punished trial, divided by the total times the participant shifts choices) and perseverative errors (two or more consecutive error responses during the reversal phase) [99]. The Probabilistic Reversal Learning task is a well-defined and ecological instrument which is widely used measuring cognitive flexibility in children [101] and adolescents [102]. Neurocognitive test instructions to children can be found in Additional file 1.

##### Child psychological symptoms

Externalising and internalising problem behaviours of the child will be assessed through parent- and teacher-ratings on the Conners’ Parent/Teacher Rating Scales-Revised: Long (CPRS-R:L [62]/CTRS-R:L [103]). We will use the subscales DSM-IV Inattentive Symptoms, DSM-IV Hyperactivity-Impulsive Symptoms, Oppositional, Anxious-Shy, Social problems and the Emotional Lability Index, resulting in questionnaires containing 44 (CPRS) and 37 items (CTRS). Items are scored on a 4-point rating scale ranging from 0 (*not true at all*) to 3 (*very much true*) with higher scores reflecting more symptoms. Both forms of the American version have a good internal consistency for all named subscales (Cronbach’s α coefficient ranging from .80 to .94) except the Emotional Lability Index, which is sufficient (Cronbach’s α coefficient ranging from .72 to .80) [103].

Symptoms of ASD will be assessed with the Social Responsiveness Scale (SRS) [104, 105] rated by parent and teacher. The 65 items can be organised into five subscales (i.e. Social Awareness, Social Cognition, Social Communication, Social Motivation, and Restricted Interests and Repetitive Behaviour) and two DSM-5 subscales (i.e. Social Communication and Interaction, and Restricted Interests and Repetitive Behaviour). Items are rated on a 4-point rating scale ranging from 1 (*not true*) to 4 (*almost always true*) with higher scores reflecting less social responsiveness. The Dutch version has a good internal consistency for both the parent- and teacher-form with Cronbach’s α coefficients of .92 and higher [106].

Brooding will be assessed using the self-report subscale of the Ruminative Response Scale (RRS; items reformulated by the authors for children from age 8) [107, 108]. Items are scored on a 4-point rating scale ranging from 1 (*almost never*) to 4 (*almost always*) with higher scores reflecting more brooding in response to sadness. The internal consistency of the American Brooding subscale is good (α = .80) when administered to adolescents [109].

##### Child well-being

We will use parents report on their child’s quality of life using the 11-item KIDSCREEN-10 [110]. Items are scored on a 5-point rating scale ranging from 1 (*never*) to 5 (*always*) with higher scores reflecting a higher health related quality of life. The internal consistency based on ten items is adequate (α = .78) [111].

##### Child mindfulness

Mindfulness skills will be assessed using self-report on the Child and Adolescent Mindfulness Measure (CAMM) [112, 113]. The CAMM consists of ten items that are scored on a 5-point rating scale ranging from 0 (*never true*) to 4 (*always true*) with lower scores reflecting better mindfulness skills. Internal consistency for the Dutch version was reasonable when administered to children (α = .71) and good for adolescents (α = .80) [112].

#### Secondary outcome measures for parents

##### Self-rated parental self-control

Adult versions of the self-control measures for children will be administered to parents. The BRIEF-A [114] is a 75-item self-report questionnaire comparable to the child version in rating scale and structure, but with slightly different subscales (i.e. Inhibit, Shift, Emotional Control, Self-Monitor, Initiate, Working Memory, Plan/Organise, Task Monitor and Organisation of Materials). The Dutch version shows a good internal consistency for the global executive composite score and both indexes (Cronbach’s α coefficient ranging from .92 to .96) and sufficient internal consistency for all subscales [114].

##### Computerised tasks of parental self-control

The parents’ version of the Stop Signal task and the Probabilistic Reversal Learning task will be identical to those described for children. The Stop Signal task shows an adequate test-retest reliability when administered to adults without a DSM-IV diagnosis in the past year (*r* = .65 for the SSRT) [115]. The Probabilistic Reversal Learning task shows adequate results when administered to adults with schizophrenia (ICC ranging from .49 to .68) [116].

As the Temporal Discounting task that will be used for children is not appropriate for use in adults, a hypothetical Temporal Discounting task will be administered to the parents. Participants make a series of choices regarding fictional amounts of money (rewards), ranging between one and hundred euros, available now versus after a specified delay interval (i.e. one month, one year, five years, ten years). Instructions are presented on the screen and are identical to those used by R. M. Hurst, H. O. Kepley, M. K. McCalla and M. K. Livermore [117]. For each trial, two variable rewards are presented in a random order, to increase the likelihood that the decisions are based on the amount in the individual trial and not on the previous trial. The participant’s score on the different delay intervals is the reward value where they switch between taking the delayed reward to taking the immediate one. The hypothetically Temporal Discounting task is widely used in various studies [97] and shows good internal consistency (α = .89) when administered to adults with and without self-reported ADHD [117]. Neurocognitive test instructions to parents can be found in Additional file 1.

##### Parental psychological symptoms

Symptoms of ADHD will be assessed using self-report on the Dutch version of the ADHD DSM-IV rating scale [118], which consists of 26 items concerning behaviour in the last two weeks (23 items) and childhood (three items). The latter three items are useful for estimating ADHD diagnosis, as onset of ADHD symptoms needs to be before the age of twelve according to the DSM-5. Items are scored on a 4-point rating scale ranging from 0 (*never or seldom*) to 3 (*very often*) with higher scores indicating more ADHD traits. The internal consistency is high for the Inattentiveness dimension (α = .83) and adequate for both the Hyperactivity (α = .75) and Impulsivity dimension (α = .72) [118].

Symptoms of ASD will be assessed using the self-rated 10-item Short Autism-Spectrum Quotient (AQ-10) [119, 120]. Each item is rated on a 4-point rating scale (1 = *definitely agree*, 2 = *slightly agree*, 3 = *slightly disagree*, 4 = *definitely disagree*) with higher scores indicating more symptoms of ASD. The internal consistency is good for the English 10-item version (α > .85) [120].

Symptoms of depression, anxiety and stress will be assessed using the self-rated 21-item Depression Anxiety Stress Scale (DASS-21) [121, 122] rated on a 4-point rating scale ranging from 0 (*did not apply to me at all*) to 3 (*applied to me very much of most of the time*). Higher scores reflect more symptoms of depression, anxiety and/or stress. All subscales of the Dutch version show a good internal consistency with Cronbach’s α coefficient ranging from .85 to .94 for students and a clinical sample of adults with an anxiety disorder and/or depression [122].

Brooding will be assessed using the Brooding subscale of the self-rated RRS [107, 108] containing five items that are rated on a 4-point rating scale ranging from 1 (*almost never*) to 4 (*almost always*). Higher scores indicate more brooding in response to sadness. The internal consistency of the Dutch Brooding subscale is sufficient (α = .78) when administered to adults [108].

##### Parental well-being

Quality of life will be assessed using the self-rated 5-item World Health Organization-Five Well-Being Index (WHO-5) [123]. Each item is scored on a 6-point rating scale ranging from 5 (*all the time*) to 0 (*not at all*). Higher scores reflect higher psychological well-being. The WHO-5 shows an adequate validity as an outcome measure in clinical controlled trials, sensitive for assessing change [124].

Emotional, psychological and social well-being will be assessed using self-report on the 14-item Mental Health Continuum-Short Form (MHC-SF) [125]. Each item is scored on a 6-point rating scale ranging from 0 (*never*) to 5 (*every day*) with higher scores indicating more positive mental health. The Dutch version shows high internal consistency for the total MHC-SF score (α = .89) and the two subscales Emotional and Psychological Well-Being (both α = .83), and adequate for the third subscale Social Well-Being (α = .74) [125].

##### Mindful parenting

We will use self-ratings on the 31-item Interpersonal Mindfulness in Parenting scale (IM-P) [126, 127] assessing 1) Listening with Full Attention; 2) Compassion for the Child; 3) Non-judgmental Acceptance of Parental Functioning; 4) Emotional Nonreactivity in Parenting; 5) Emotional Awareness of the Child and 6) Emotional Awareness of Self. Items are scored on a 5-point rating scale (1 = *never true*, 2 = *rarely true*, 3 = *sometimes true*, 4 = *often true*, 5 = *always true*) with higher scores reflecting more mindful parenting. The Dutch version of the IM-P shows a good internal consistency based on 29 items (α = .89) [126].

#### Tertiary measures for children

##### Self-rated

We will use the 8-item subscale Body Awareness of the Body Experience Questionnaire for Children (BEQC) as a tertiary measure, which shows an adequate internal consistency for the Dutch version (α = .71) [128]. Sensory-processing sensitivity will be assessed using the 12-item Highly Sensitive Child Scale (HSCS). The HSCS shows an adequate internal consistency (α = .79) [129]. For children aged eleven and older we will use the 24-item Inventory of Callous-Unemotional Traits (ICU) assessing uncaring, callousness and unemotional traits. The Dutch version shows a good internal consistency (α = .89) [130]. Self-compassion will be assessed by using one item per subscale of the Self-Compassion Scale [131] with the highest factor loading [132]. This resulted in six exploratory items which were reformulated by the authors for children. Mind-wandering will be assessed using an exploratory questionnaire (items developed by JB for this study, 2015) containing three items (e.g. “When you are busy with schoolwork, a task or a chore, do you notice that you are daydreaming?”).

##### Parent-rated

The 18-item DSM-IV-based Strengths and Weakness of ADHD Symptoms and Normal Behaviour (SWAN) [133] will be used to assess ADHD traits (inattentiveness and hyperactivity-impulsivity). The Dutch version of the SWAN shows a good internal consistency (α > .87) when administered to parents (α = .88) [134] and will be included as it provides good resolution across the full range of continuous ADHD traits, thereby potentially providing additional information to the Conners’ Rating Scales [135]. Neuroticism, as a covariate for the HSCS, will be assessed by administering the 5-item Big Five questionnaire [136]. Sleeping habits will be assessed using the 5-item Sleep Questionnaire used in standard clinical care assessing problems with sleeping in, sleeping through and total amount of sleep compared to children of the same age.

##### Teacher-rated

The above described 18-item SWAN [133] will be administered to teachers, also showing a high internal consistency for the Dutch version (α = .91) [134].

##### Tasks

Reading speed and accuracy will be assessed with the Dutch One Minute Reading Test; a test of word decoding, in which children are instructed to read as many words from a list as possible within one minute. The test shows a good test-retest reliability with high correlations ranging from .80 to .92 [137].

#### Tertiary measures for parents

##### Self-rated

The 12-item Highly Sensitive Person Scale (HSPS) will be administered to parents assessing sensory-processing sensitivity, showing a good internal consistency (α = .89) when administered to adults [136]. Neuroticism, as a covariate for the HSPS, will be assessed by administering the 5-item Big Five questionnaire [136]. For the six subscales of the Self-Compassion Scale, the item that correlates the most with the total scale will be selected to create a 6-item questionnaire to explore self-compassion [132].

### Qualitative research

Qualitative interviews with families and mindfulness teachers will be conducted after the family MBI, with the aim to capture the richness and heterogeneity of experiences of families with ADHD potentially not tapped by the quantitative assessments, and to facilitate adaptations to the MYmind protocol based on participant and mindfulness teacher experience. Purposive sampling will be used to include a subset of families with different backgrounds, age groups, gender and drop-outs will also be interviewed. Data will be generated until saturation is reached. The interviews are semi-structured using a topic guide focusing on two main topics: 1) facilitators and barriers to participating in family MBI training; and 2) effects of family MBI on parent, child and parent-child interactions. Interviews will be conducted separately with parent, child and mindfulness teachers. All interviews will be audio recorded, transcribed verbatim and analysed in the Atlas.ti software using Grounded Theory [138]. Analysis will be performed by a team of researchers to ensure no data get lost. Essential aspects of qualitative research concerning relevant participant selection, appropriate data collection methods, comprehensive data collection process and data analysis will be followed [139].

### Biological assessments

Studying (epi)genetic factors alongside environmental factors in relation to family MBI can help understand the biological basis of family MBI. Five saliva samples will be drawn from children for DNA and RNA isolation and assessment of biomarkers: one OG-500 DNA at baseline for genotyping of DNA; one Oragene OG-575 DNA at baseline and one at end of treatment for methylomic profiling of CpG DNA sequences; and one Oragene RE-100 RNA at baseline and one at end of treatment for profiling of transcriptomics (mRNA), microRNA expression and epigenomic profiling. The collection of saliva during end of treatment will be scheduled during the same time of the day as the collection at baseline within participants to account for hormonal fluctuations. Children will be asked to refrain from food and drinks (except water) and cigarettes thirty minutes before collection. Samples will be processed under barcode in a validated and well-controlled pipeline designed to process samples for clinical use. The material of biological nature will be stored at − 20 or − 80 °C (as appropriate; RE-100 RNA vials to be stored at − 80 °C to minimise any RNA degradation). Data will be stored in an automated, validated laboratory information system (Labvantage: www.labvantage.com/).

### Procedure and treatment allocation

Recruitment will take place within the outpatient clinics of Karakter through screening by the researchers of currently treated patients or through referral by the psychiatrist. In addition, recruitment will take place via media advertisement (e.g. flyers and website). In case there are no contra-indications (i.e. exclusion criteria, crisis situation or family does not want to be approached for scientific research), families will be contacted by phone to inform them about the study and to establish whether they meet the inclusion criteria. Next, information letters will be sent to parent and child via e-mail. Families will have a minimum of two weeks to consider participation. Families willing to participate will then be sent forms of informed consent in the post: parental consent of child participation, parental consent of own participation, child consent (age > =12 years) of own participation. Participants not referred by Karakter will also give consent to request medical data from the institute/clinician who diagnosed ADHD. The participants will be randomised after completion of the informed consent forms. In case families withdraw consent before the baseline assessments, randomisation will be undone and participants can be replaced, in order not to let non-participants imbalance the treatment groups for the primary analyses. School teachers and general practitioners of included participants will be informed about the study via telephone, email and/or postal letter. The school teachers will be requested not to ask the family about treatment allocation to keep them ‘blinded’. Likewise, the participants will be requested not to speak about treatment allocation to the school teachers.

**Fig. 1:**
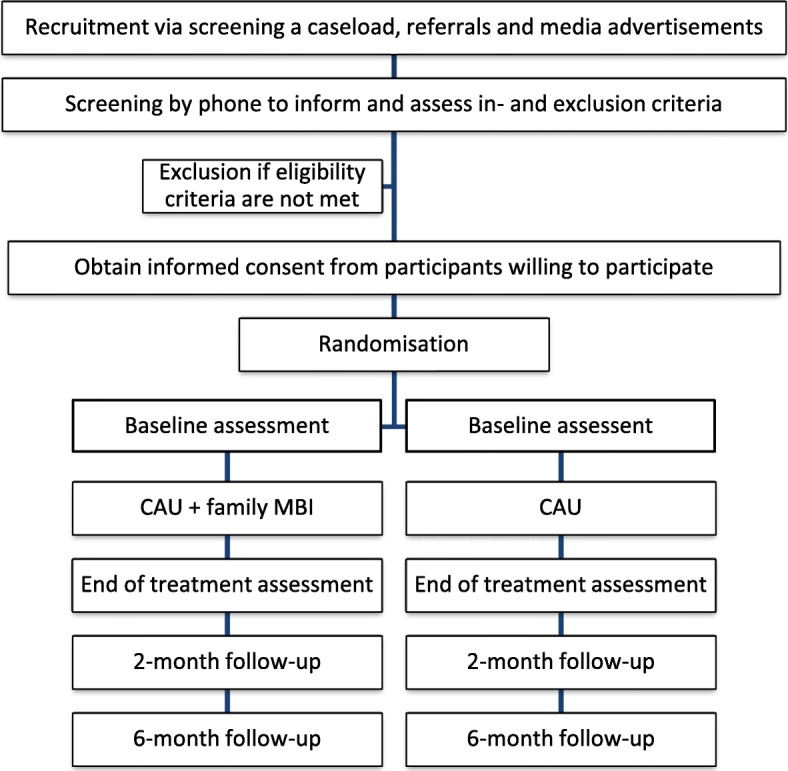
Recruitment and study procedure *Note*. CAU=Care-As-Usual, MBI = Mindfulness-Based Intervention

Assessments will take place at baseline (T0), end of treatment (T1), two (T2) and six (T3) months after end of treatment. For the family MBI condition, T0 will be scheduled in the two weeks prior to the start of the intervention and T1 in the two weeks after the 8th session of the MYmind training. The booster session will be followed by the first follow-up assessments (T2) which will be two months after T1 and the second follow-up assessments (T3) will be scheduled half a year after T1. Intervals between assessments will be kept similar for the family MBI and CAU condition. For baseline, end of treatment and 6-month follow-up assessments, participants will be invited to a location of Karakter. Assessments will be conducted by a researcher or research assistant. Participants will be asked to interrupt ADHD medication intake 48 h prior to these assessments. For the 2-month follow-up assessments, both parent and child will receive an invitation to complete questionnaires online (not at Karakter). School teachers will be asked to complete all questionnaires online at baseline, end of treatment and 2-month follow-up. See Table 1 for which assessments for children will be taken at which time points and Table 2 for assessments for parents and time points. Further, see Fig. 1 for a flowchart on recruitment and the study procedure.

**Table 1:** Assessments for children and time points

Assessments for children	Time points			
*Descriptives*				
Demographics	T0			
WISC-III (Vocabulary and Block Design)	T0			
Investigator-rated K-SADS-PL	T0			
Self-rated PDS	T0			T3
*Primary outcome measure*				
*Parent-rated child self-control*				
BRIEF-P	T0	T1	T2	T3
*Secondary outcome measures*				
*Teacher-rated self-control*				
BRIEF-T	T0	T1	T2	
*Computerised tasks of self-control*	T0	T1		T3
*Psychological symptoms*				
Parent-rated CPRS-L:R (subscales DSM-IV inattentive symptoms, DSM-IV hyperactivity-impulsive symptoms, oppositional, anxious-shy, social problems, emotional lability index)	T0	T1	T2	T3
Teacher-rated CTRS-L:R (subscales DSM-IV inattentive symptoms, DSM-IV hyperactivity-impulsive symptoms, oppositional, anxious-shy, social problems, emotional lability index)	T0	T1	T2	
Parent-rated SRS	T0	T1		T3
Teacher-rated SRS	T0	T1		
Self-rated RRS (Brooding)	T0	T1		T3
*Well-being*				
Parent-rated KIDSCREEN-11	T0	T1		T3
*Mindfulness*				
Self-rated CAMM	T0	T1	T2	T3
*Tertiary measures*				
*Self-rated*				
BEQC	T0	T1		T3
HSCP	T0			
ICU (ages 11+)	T0	T1		
Self-compassion (exploratory items)	T0	T1		T3
Mind-wandering (exploratory items)	T0	T1	T2	T3
*Parent-rated*				
SWAN (optional)	T0	T1		T3
Neuroticism (covariate for HSCP)	T0			
Sleeping habits	T0	T1		T3
*Teacher-rated*				
SWAN	T0	T1		
*Tasks*				
One Minute Reading Test	T0	T1		T3
*Saliva collection*	T0	T1		

T0 baseline, T1 end of treatment, T2 2-month follow-up, and T3 6-month follow-up, WISC-III Wechsler Child Intelligence Scale-Third Edition, K-SADS-P Schedule for Affective Disorders and Schizophrenia for School-Age Children-Present and Lifetime Version, PDS Pubertal Development Scale, BRIEF-P Behaviour Rating Inventory of Executive Function-Parent form, BRIEF-T Behaviour Rating Inventory of Executive Function-Teacher form, CPRS-L:R Conners’ Parent Rating Scales-Revised: Long, CTRS-L:R Conners’ Teacher Rating Scales-Revised: Long, SRS=Social Responsiveness Scale, RRS = Ruminative Response Scale, CAMM = Child and Adolescent Mindfulness Measure, BEQC=Body Experience Questionnaire for Children, HSCP=Highly Sensitive Child Scale, ICU=Inventory of Callous-Unemotional Traits, and SWAN=Strengths and Weakness of ADHD Symptoms and Normal Behaviour

**Table 2:** Assessments for parents and time points

Assessments for parents	Time points			
*Descriptives*				
Demographics	T0			
WAIS-III (Vocabulary and Block Design)	T0			
Self-rated K10	T0			
Investigator-rated MINI	T0			
*Secondary outcome measures*				
*Self-rated self-control*				
BRIEF-A	T0	T1	T2	T3
*Computerised tasks of self-control*	T0	T1		T3
*Psychological symptoms*				
Self-rated ADHD DSM-IV rating scale	T0	T1	T2	T3
Self-rated AQ-10	T0	T1		T3
Self-rated DASS-21	T0	T1		T3
Self-rated RRS (Brooding)	T0	T1		T3
*Well-being*				
Self-rated WHO-5	T0	T1		T3
Self-rated MHC-SF	T0	T1		T3
*Mindful parenting*				
Self-rated IM-P	T0	T1	T2	T3
*Tertiary measures*				
Self-rated HSPS	T0			
Self-rated neuroticism (covariate for HSPS)	T0			
Self-rated self-compassion (exploratory items)	T0	T1		T3

*Note*. T0 baseline, T1 end of treatment, T2 2-month follow-up, and T3 6-month follow-up, WAIS-III=Wechsler Adult Intelligence Scale-Third Edition, K10 = 10-item Kessler Psychological Distress Scale, MINI = Mini-International Neuropsychiatric Interview, BRIEF-A = Behaviour Rating Inventory of Executive Function-Adult version, AQ-10 = 10-item Short Autism-Spectrum Quotient, RRS = Ruminative Response Scale, WHO-5 = 5-item World Health Organization-Five Well-Being Index, MHC-SF = Mental Health Continuum-Short Form, DASS-21 = 21-item Depression Anxiety Stress Scale, IM-P=Interpersonal Mindfulness in Parenting Scale, and HSPS=Highly Sensitive Person Scale

### Sample size

#### Power calculation

Power analyses are based on the primary hypothesis that family MBI, relative to control, will lead to improvement in self-control (as measured by the BRIEF) at end of treatment. The BRIEF has shown modest to large effect sizes in MBI studies for clinical samples, including bipolar disorder [140][Cohen’s d effect sizes from 0.08–1.33 on self-report BRIEF] and adult ADHD [57][Cohen’s d effect size from 0.43 to 0.93 on self-report BRIEF]; [56][Cohen’s d effect size from 0.67 to 1.72 on self-report BRIEF]. Further, in previous work using the MYmind programme (unpublished data on a larger non-randomised waitlist-controlled pilot in youth with ADHD *N* = 195 families using parent-report BRIEF; see also [55][Cohen’s d effect size from 0.0 to 1.8 on parent-report BRIEF; 0.2 to 0.5 on teacher-rated BRIEF]), a moderate effect on the BRIEF was found. Therefore power calculations were based on a moderate effect size of 0.4. Power analyses were run using the software G*power 3.1 and based on the procedure by G. F. Borm, J. Fransen and W. A. Lemmens [141]. Using this method, the N per group is obtained from the N per group for t-test multiplied by the design factor (D). The design factor D = 1–r^2^, where r^2^ is the correlation between baseline and end of treatment on the BRIEF. We determined r using the data of a normative sample over a comparable time frame (six weeks): .86 [142]. As the period between baseline and end of treatment is slightly longer in this trial (around eleven weeks) the power analyses was conducted using a more conservative estimate of *r* = .76, hence D = 1–.76^2^ = .42.

Based on a power of 80%, a two-tailed test with α = .05 and an estimated effect size of 0.4, we need 100*.42 = 42 children in each group. Past studies conducted using the MYmind programme (see section *Intervention*) using non-randomised designs in youth with ADHD and ASD found attrition rates from 5 to 15%. Therefore, we will recruit a slightly larger sample (2xN = 50, total *N* = 100), allowing room for attrition of around 15%.

### Randomisation

Randomisation by means of minimisation [143] will be performed after completion of the informed consent forms. The treatment group that most strongly minimises the imbalance between the two conditions is chosen to allocate the patient. To balance possibly prognostic factors across treatment groups, the following procedure will be performed: first, a stratification for age group is performed (elementary school or secondary school). Then, block randomisation is performed (with varying predefined block sizes), combined with minimisation. The three minimisation factors are: 1) medication for ADHD at baseline (medication versus no medication); 2) gender (male versus female); and 3) age (younger (child: age 8–10 or adolescent: age 12–14) versus older (child: age 10–12 or adolescent: age 14–16)). Minimisation factors are of equal importance, and block randomisation always wins over minimisation. Concealment of allocation is guaranteed as the sequence behind the randomisation will be unknown by the executing researcher.

### Data collection, management analysis

As soon as the participant is enrolled, across all assessments he or she will only be identifiable via a unique pseudocode identifier to anonymise all data. A separate protected data-base will link the unique pseudocode to the participants’ names. Anonymous and non-anonymous (e.g. informed consent forms) data will be stored in separate password protected folders. Video-data of the sessions will be transported with an encrypted external hard disk and stored on a network attached storage only accessible for the appointed researchers. Questionnaire data will be collected and stored with the online electronic data capture software CASTOR EDC [144], which tracks and logs any manual changes made to raw data and is fully Good Clinical Practice (GCP) compliant. Quantitative data that is first collected on paper (e.g. IQ test, diagnostic interview) will be manually entered in CASTOR EDC and the entry will be double checked by another researcher. To prevent missing data, the force step completion function will be used so informants can only continue with the questionnaire when all items are completed. When questionnaires are not completed within the expected period, participants/school teachers will be sent online reminders. If no response follows they will be contacted per telephone to discuss the absence of response and to motivate them to complete the questionnaires, if necessary with concessions (e.g. fewer questionnaires). In case participation is discontinued, reasons will be noted and participants will be asked to complete a drop-out questionnaire with the main outcome measures (BRIEF-P, BRIEF-A, CPRS and parental ADHD self-report). Clinically informative results derived from child- and parent-rated questionnaire data will be reported by the researchers and sent to the involved participant after study participation ends.

### Statistical analyses

Data will be analysed and reported according to CONSORT guidelines. Baseline demographic and clinical characteristics of the family MBI- and CAU-groups will be compared to examine whether these have been distributed evenly across the two groups by the randomisation. The primary analyses will be aimed at comparing results on the outcome measures at end of treatment between the family MBI and CAU condition, controlling for baseline levels and possible other baseline differences between intervention and CAU groups. To test our primary hypothesis that assignment to family MBI (relative to CAU) will improve child self-control, we will use ANCOVA with group condition (family MBI versus CAU) as the primary independent variable, and child self-control (continuous BRIEF-P score) at end of treatment as the primary dependent variable and child self-control at baseline as covariate. Cohen’s d effect sizes will be calculated. Analyses will be conducted both according to intention-to-treat principle, and per-protocol on completers (family MBI condition: child and parent attended > = 4 family MBI sessions). Intention-to-treat analyses will be conducted using available case analysis (not considering individuals with missing data) as well as imputation, as described in [145]. In case both parents participate in the assessments, data of the parent with the most complete dataset will be used for the ANCOVA, and if both are complete, the sex of the parent (or age, for same-sex parents) will determine which dataset will be used so that the fathers:mothers ratio is most similar across both study arms.

Secondary analyses, aimed at examining the consolidation of treatment effects at follow-ups, will use multilevel modelling with time point as repeated measurement, following the approach described in [58]. Moreover, we will conduct a set of complementary analyses to derive prediction models for response to intervention from baseline characteristics, and explore interactions between child and parental outcomes.

### Monitoring

A data protection officer independent from the researcher will be assigned and monitors 1) the protection of the rights and well-being of the participants; 2) whether the reported research data is accurate and completely verifiable in source documents; and 3) whether the implementation of the research is consistent with the approved protocol/amendment(s) at that time, with GCP and the applicable legal requirements [146]. After including the first five participants, all data will be monitored. That is, the accuracy and completeness of all data (on item/trial level) per participant will be checked. After including participant five to ten, the completeness and accuracy of the data on summary/scale level will be checked for all participants. Thereafter, data on summary/scale level will be checked randomly for one in five participants. Outcomes of this data monitoring will be summarised in an overview and reported to all research personnel.

Based on previous studies on MBI for children and Mindful Parenting, deterioration as a result of family MBI is not expected. Therefore, no interim analysis will be conducted. Adverse events (AEs) are defined as any undesirable experience occurring to a participant during the study, whether or not considered related to the family MBI. All AEs reported spontaneously by the participant or observed by the researcher or mindfulness teachers will be recorded. All serious adverse events (SAEs, resulting in death, life threatening, requiring hospitalisation or other important medical events) will be communicated to the principal and coordinating researchers, who will report the SAEs to the accredited ethics committee that approved the protocol, within fifteen days after the sponsor has first knowledge of the SAE. SAEs that result in death or are life threatening should be reported expedited. The expedited reporting will occur not later than seven days after the responsible researcher has first knowledge of the SAE.

## Discussion

Self-control is a malleable determinant of success in health, wealth, parenting, and avoiding crime [147]. Hence, improving self-control in children with self-control deficits has an important impact on their life and society. An example of a clinical population in which targeting self-control in treatment is pressing is youth with ADHD. ADHD is associated with adverse outcomes including impediment on academic achievement, mental and substance use disorders, criminality, and employment [148]. Current CAU for youth with ADHD is often not sufficient in improving self-control. Furthermore, CAU is generally not focused on mental health and well-being of the parents, although this has impact on the (treatment of the) child as well. ADHD medication can have undesired side effects, is refused by some families, compliance may be low, and improvements do not last after medication discontinuation [35]. These shortcomings might be addressed by offering a family-based MBI in addition to CAU. Self-regulation is present at the basis of MBIs and work by cognitive neuroscientists demonstrates that brain structures and functions that are involved in self-control are altered with (mindfulness) meditation. There is strong evidence of positive overall effects of MBIs in children as well as adults [21, 149, 150]. However, studies investigating the effects of MBIs on self-control in youth with ADHD and/or their parents are scarce and there is a need for methodologically stronger trials.

This protocol describes an adequately powered RCT studying family MBI as an innovative non-pharmacological approach in the treatment of youth with ADHD. Children with comorbidities (e.g. ASD, ODD, dyslexia) will be included which has the advantage of increasing the representativeness of the sample for the clinical ADHD population. Where previous studies were uncontrolled or waitlist controlled, the current trial allows comparison with a CAU control group. Actually received CAU prior to and during study participation will be registered in both the control and intervention condition. The intervention is a manualised family MBI (MYmind) for children and their parents given by well trained experienced mindfulness teachers who will be evaluated in terms of both their adherence to the protocol and competence. Another strength of the study is that assessments will be done with different informants allowing taking account of rater effects. Questionnaires will not only be rated by self and parents, but also by teachers who are not involved in the intervention. Next to subjective ecologically valid questionnaires, objective computerised tasks will be administered to explore effects on different aspects of self-control. Further, a broad range of outcomes (e.g. neuropsychological functions, clinical symptoms, positive health) will be assessed in both the child and the parent. This not only allows studying the effect of family MBI on child and parental outcomes but also how they relate to each other. This will increase our understanding of the influence of parental symptoms, functioning and well-being on the child and vice versa, and the possible role that MBI on a family level may play in targeting child and/or parental needs. In addition, follow-up assessments until six months after the end of treatment make it possible to investigate both the short- and long-term effectiveness of the intervention. In a similar trial of an MBI in adults with ADHD, significant effects on self-control assessed with the BRIEF were only found at follow-up [58], as it might take more time and practice before MBI results in improvement of real-life self-control skills. Effects of self-control are suggested to follow a continuum, therefore interventions that achieve even small improvements in self-control for individuals, could shift outcomes across the population as a whole in a positive direction to impact health, wealth and crime rates [1]. Finally, ADHD is one of several psychopathologies (e.g. ODD, CD, addictions, mood- and anxiety disorders) that involve self-control deficits. Hence, results of the proposed RCT are in a cross-disorder perspective informative for a broad clinical population.

## Additional file

Additional file 1: Neurocognitive tests instructions. Neurocognitive test instructions (translated from Dutch). (DOCX 18 kb)

## Abbreviations

ADHD: Attention Deficit/Hyperactivity Disorder
AE: Adverse Event
AQ-10: Short Autism Spectrum Quotient
ASD: Autism Spectrum Disorder
BEQC: Body Experience Questionnaire for Children
BRIEF: Behaviour Rating Inventory of Executive Function
CAMM: Child and Adolescent Mindfulness Measure
CAU: Care-As-Usual
CD: Conduct Disorder
CEQ: Credibility/Expectancy Questionnaire
CMO: Central committee on research involving human subjects
CPRS-R:L: Conners’ Parent Rating Scales-Revised: Long
CTRS-R:L: Conners’ Teacher Rating Scales-Revised: Long
DASS-21: Depression Anxiety Stress Scale
DSM-5: Diagnostic and Statistical Manual of Mental Disorders, Fifth Edition
DSM-IV: Diagnostic and Statistical Manual of Mental Disorders, Fourth Edition
GCP: Good Clinical Practice
HSCS: Highly Sensitive Child Scale
HSPS: Highly Sensitive Person Scale
ICU: Inventory of Callous-Unemotional traits
IM-P: Interpersonal Mindfulness in Parenting scale
IQ: Intelligence Quotient
K10: Kessler Psychological Distress Scale
K-SADS-PL: Schedule for Affective Disorders and Schizophrenia for School-Age Children-Present and Lifetime Version
MBCT: Mindfulness-Based Cognitive Therapy
MBI: Mindfulness-Based Intervention
MBSR: Mindfulness-Based Stress Reduction
MHC-SF: Mental Health Continuum-Short Form
MINI: Mini-International Neuropsychiatric Interview
ODD: Oppositional Defiant Disorder
PDS: Pubertal/Physical Development Scale
RCT: Randomised Controlled Trial
RRS: Ruminative Response Scale
SAE: Serious Adverse Event
SPIRIT: Standard Protocol Items: Recommendations for Interventional Trials
SRS: Social Responsiveness Scale
SSRT: Stop Signal Reaction Time
SWAN: Strengths and Weakness of ADHD Symptoms and Normal Behaviour
TAC: Teaching Assessment Criteria
TiC-P: Trimbos and iMTA questionnaire on Costs associated Psychiatric illness
VMBN: Association of mindfulness-based teachers in the Netherlands and Flanders
WAIS-III: Wechsler Adult Intelligence Scale-Third Edition
WHO-5: World Health Organization-Five Well-being Index
WISC-III: Wechsler Intelligence Scale for Children-Third Edition
