# Supplementary material for: Mindfulness for children with ADHD and Mindful Parenting (MindChamp): Protocol of a randomised controlled trial comparing a family Mindfulness-Based Intervention as an add-on to care-as-usual with care-as-usual only
Source: BMC Psychiatry. 2018 Jul 25;18:237. doi: 10.1186/s12888-018-1811-y (PMC6060473; doi:10.1186/s12888-018-1811-y)
Supplement: Supplementary file 1 — Neurocognitive tests instructions. Neurocognitive test instructions (translated from Dutch). (DOCX 18 kb) [file 12888_2018_1811_MOESM1_ESM.docx]

**Neurocognitive tests instructions (translated from Dutch)**

***Stop Signal task – parent and child***

In this task you will see an X or an O in the centre of the screen. When you see the X press the left button, that is this button. When you see the O, press the right button, that is this button. Respond as quickly as you can without making any mistakes. You must keep both index fingers on the buttons. Always keep your fingers on the buttons, then you will be able to respond as quickly as possible.

But beware: sometimes you will hear a beep just after the appearance of the X or O, then you should not press one of the buttons. Sometimes you will, but sometimes you will not succeed in not pressing one of the buttons. That is the case with everyone. Some people only press a button after they have not heard the beep. That is not the intention! You are not supposed to wait for the beep. It remains important to press one of the buttons as soon as possible when you see the X or the O.

***Temporal Discounting task - child***

With this task you can collect money, which you can take home with you!

You will soon see two planes on the screen, which are flown by two different pilots: Mikel and Anouk. They carry money with them in their plane. You can see how much money they carry by counting the pennies, or by looking at this number.

One plane always flies on the left, and one always on the right. You have to choose one plane. You have to press the ‘X’ with your left hand to choose the plane on the left side, and press the ‘O’ with your right hand to choose the plane on the right side. The money from the plane of your choice will end up in your basket. The planes carry a maximum of five cents, like this one.

Mikel is afraid of great heights and always flies just above the ground. Anouk flies on every altitude she wants, and that is usually higher than Mikel. If Anouk is flying high and Mikel just above the ground and both planes drop their money at the same time, of which plane the money will reach your basket earlier?

I will explain a few more things and then you will practice. When you practice, the money you collect is not really yours. In the real game you play for real money, which you can take home after this visit.

Anouk can fly at six different levels. The higher her plane flies, the longer it will take for the money to reach your basket.

Anouk is now going to fly on different levels. Pay attention to the height at which Anouk flies and to how long it takes for the money to reach your basket. We are going to practice this now. Are you ready?

Now you know how long it takes for the money to reach your basket. I will further explain how the game works.

During the game, you will see Mikel and Anouk’s planes on the screen, one on the left side and one on the right side. Anouk and Mikel can fly on both sides of the screen. You then choose which plane you want the money from: you press this button for the plane on the right side, and this button for the plane on the left side.

If you choose the money of Mikel’s plane, the money will reach your basket right away because he always flies at the lowest level. Anouk usually flies higher than Mikel. If you choose the money from Anouk’s plane, you will usually have to wait longer for the money to reach your basket. As soon as the money reached your basket, the planes will immediately fly a new mission. So you will see the planes again right away.

I am going to give you a tip now. The plane of Mikel usually carries less money on board than the plane of Anouk! Mikel can carry one, two, three or four cents on board, while the plane of Anouk always carries five cents. So if you choose Mikel’s plane, you will usually get a little less money straight away. If you choose Anouk’s plane, you will usually have to wait longer, but get more money. Shall we try? Are you ready?

Good job! Now you have practiced, you are ready for the real task. As I said earlier, the practice trials were not for real money, but in a moment the real game starts and then the money you collect is really yours. The planes fly forty missions so you can collect money forty times. With some tasks it is important to press a button as quickly as you can. In this task, speed is not important. You can take all the time you want to decide which plane you want to choose.

***Temporal Discounting task -parent***

You will be asked to make some choices about money. You will not actually get the money that you choose, but make your choices as though you were really going to get the money. When you have made a choice, a new set of monetary amounts will be presented. This process will be continued until the end of the task.

Make your choice by pressing the left button to choose the amount on the left side and the right button to choose the amount on the right side. There are no right or wrong answers. We would like you to choose what you would choose if you imagine you would actually receive the money.

***Probabilistic Reversal Learning task – parent and child***

In this task you will repeatedly see two identical minions, one red and one yellow *(for each time point different colours are used)*, as you can see on this picture. One of the images is more often correct than the other and you are supposed to choose the image of which the computer most often says it is correct. If you choose the image in the upper block press this button, if you choose the left block press this button, if you choose the right block press this button and if you choose the bottom block press this button. You must try to find out yourself which image is correct and which is false. At certain moments the rule can change, so that the other image suddenly becomes more often correct. Then switch image. This can happen one or more times during the task. But beware: sometimes the computer indicates that the image is false, while it was actually the correct choice. Keep on trying to find out which image is the correct choice.
